# Effect of Cis Acting Potential Regulators in the β Globin Gene Cluster on the Production of HbF in Thalassemia Patients

**DOI:** 10.4084/MJHID.2013.012

**Published:** 2013-02-16

**Authors:** Pooja Dabke, Roshan Colah, Kanjaksha Ghosh, Anita Nadkarni

**Affiliations:** National Institute of Immunohaematology (ICMR), 13th Floor, New Multistoried Building, K.E.M. Hospital Campus, Parel, Mumbai –4000 12.

## Abstract

The clinical presentation of β-thalassemia intermedia phenotypes are influenced by many factors. The persistence of fetal hemoglobin and several polymorphisms located in the promoters of γ- and β-globin genes are some of them. The aim of this study was to evaluate the combined effect of the −158 Gγ (C→T) polymorphism and of the (AT)x(T)y configuration, as well as their eventual association with elevated levels of HbF in β-thalassemia carriers, β-thalassemia intermedia, β-thalassemia major and normal controls of Indian origin. The −158 Gγ T allele was found to be associated with increased levels of HbF in β thalassemia carriers, and not in wild-type subjects. In the homozygous group, the −158 Gγ T allele was significantly higher in the thalassemia intermedia group (66%) as against the thalassemia major group (21%). The (AT)9(T)5 allele did not show any association with raised HbF levels. However 24% of milder cases showed presence of this allele. This study suggests that two regions of the β globin cluster, whether in cis or in trans to each other, can interact to enhance HbF expression when a β thalassemic determinant is present in heterozygosity and help in amelioration of the severity of the disease in homozygotes.

## Introduction

The synthesis of fetal hemoglobin (HbF) in normal adults persists at very low levels and is confined to a small subset of erythrocytes called the F cells. HbF makes up <1% of the total hemoglobin. However some genetic conditions like hereditary persistence of fetal hemoglobin (HPFH) that are caused by point mutations in the promoter region of γ globin gene or large deletions of the β globin gene cluster, show increase in the HbF level even in adult life. There are several reports indicating that a number of polymorphic markers in the β globin cluster contribute to increase in the fetal Hb levels under erythropoietic stress.^1^,^2^ One of the genetic determinants that is thought to cause a modest increase in HbF level is the C→T substitution at −158 of the Gγ globin gene (*XmnI* polymorphism). Some reports documented that this polymorphism has strong association in thalassemia and sickle cell anemia patients, showing high expression of Gγ chains rather than high HbF levels.^3^–^5^ A polymorphic repeat (AT)_x_(T)_y_ at −530 of the β globin gene has shown to play an important role in β globin gene regulation. The (AT)_9_(T)_5_ repeats affect promoter activity due to the presence of a binding site for the repressor protein BP1. This configuration [(AT)_9_(T)_5_] has also been associated with high HbF levels in thalassemia intermediates and sickle homozygotes.^6^–^8^

In this study we have tried to look at the combined effect of these two polymorphisms on HbF levels in β thalassemia carriers and homozygotes.

## Materials and Methods

### Hematological Studies

Peripheral blood samples were collected after informed consent from 50 β thalassemia homozygote individuals, 45 carriers of β thalassemia and 50 normal controls. Red cell indices were measured on an automated blood cell counter (Sysmex K 1000). HbA_2_ and HbF levels were measured using cation exchange HPLC on the Variant Hemoglobin Testing System (Bio-Rad Laboratories, Inc., Hercules, CA, USA).

### Molecular analysis

Genomic DNA was isolated from peripheral blood leucocytes using the QIA amp Blood Mini Kit. β thalassemia mutations were characterized by reverse dot blot hybridization^9^ or amplification refractory mutation system (ARMS).^10^ The uncharacterized mutations were identified by automated DNA sequencing on the ABI Prism 310 sequencer. *XmnI* polymorphism was studied using PCR–RFLP method.^11^ The Gene Scan Analysis program on the ABI Prism 310 DNA Sequencer was used to study the (AT)_x_(T)_y_ motifs at the −530 region upstream of the 5′ β-globin gene.

### Statistical analysis

Continuous variables were expressed as mean ± SD. The *X*^2^test was used for the categorical variables as needed. Statistical significance was set at p<0.05.

## Results

Among our 45 heterozygotes, HbA2 levels ranged from 4.1 % to 6.3 % (mean ± SD 4.7± 0.77 %) HbF levels ranged from 0.2 % to 3.4% (mean ± SD 1.5± 0.90 %). Thirteen of these subjects had HbF levels more than 1.2%. Of these 80% of cases showed the presence of the *XmnI* polymorphism. The heterozygotes showing homozygosity for the *XmnI* polymorphism (+/+) showed the highest range for HbF levels (2.2%–3.4%, mean± SD 2.7± 0.45 %). 10 different β thalassemia mutations were seen among this group. 44% of the individuals showed the presence of the IVS1nt5 (G→C) mutation which is the commonest mutation reported in the Indian population. The analysis of (AT)_x_(T)_y_ motif showed 3 different configurations- (AT)_7_(T)_7_, (AT)_9_(T)_5_, (AT)_8_(T)_9_ which gave rise to six different genotypes. 22.2% of the β thalassemia chromosomes were linked to the (AT)_9_(T)_5_ motif. 5% of these (AT)_9_(T)_5_ chromosomes showed moderate increase in HbF levels. Table 1 shows the association of different motifs at the −530 region with various β thalassemia mutations. The 63% of the chromosomes carrying the IVS1nt 5 (G→C) mutation showed the presence of (AT)_8_(T)_9_ motifs, while 67% of the chromosomes with the IVS1nt 1 (G→T) mutation were associated with the (AT)_7_(T)_7_ motif. All the 4 chromosomes carrying the Codon 15(G→A) mutation were linked to the (AT)_9_(T)_5_ motif.

The hematological and molecular data of the β thalassemia homozygotes is shown in Tables 2 and 3. In the thalassemia major group, age at presentation varied from 6 months to 1.5 yrs and in the thalassemia intermedia group it varied from 2 yrs to 31 yrs. Out of the 50 homozygotes included in the study, 35 were on regular transfusion (β thalassemia major), while 15 were untransfused or required intermittent transfusions (β thalassemia intermedia). The HbF level in the transfusion independent cases ranged from 53.8%–98.3%. In the β thalassemia major group the HbF levels at presentation ranged from 37.8 % to 99.3%. The presence of the *XmnI* T allele showed a statistically significant difference among the thalassemia intermedia cases (66%) versus the thalassemia major cases (21%) (p<0.001). The (AT)_9_(T)_5_ motif was predominantly found in the transfusion independent individuals (27%) as against the transfusion dependent ones (14%). 9 different β thalassemia mutations were seen among the β thalassemia homozygous group. 64% of the cases showed the presence of the IVS1nt5 (G→C) mutation. The milder β thalassemia mutations like Cap site +1 (A→C) and −88 (C→T) were seen only in the β thalassemia intermedia group.

Among the 50 normal controls the HbF levels ranged from 0.1% to 0.8%. From this group, 42% of the chromosomes showed the presence of *XmnI* polymorphism. The (AT)_7_(T)_7_ motif was reported in 80% of chromosomes followed by the (AT)_9_(T)_5_ in 20 % of chromosomes.

## Discussion

The clinical manifestations of β thalassemia intermedia phenotypes are influenced by the persistence of fetal hemoglobin and by several polymorphisms located in the promoters of the γ- and β**-**globin genes. The association of a moderate increase in HbF, with certain polymorphic configurations in the Gγ-Aγ- ϕβ region of the β globin cluster, has not always been straightforward. In the so-called Swiss type of HPFH where the HbF level is moderately high, it has been difficult to assess the contribution of the β globin cluster-dependent factors.

**Figure 1:**
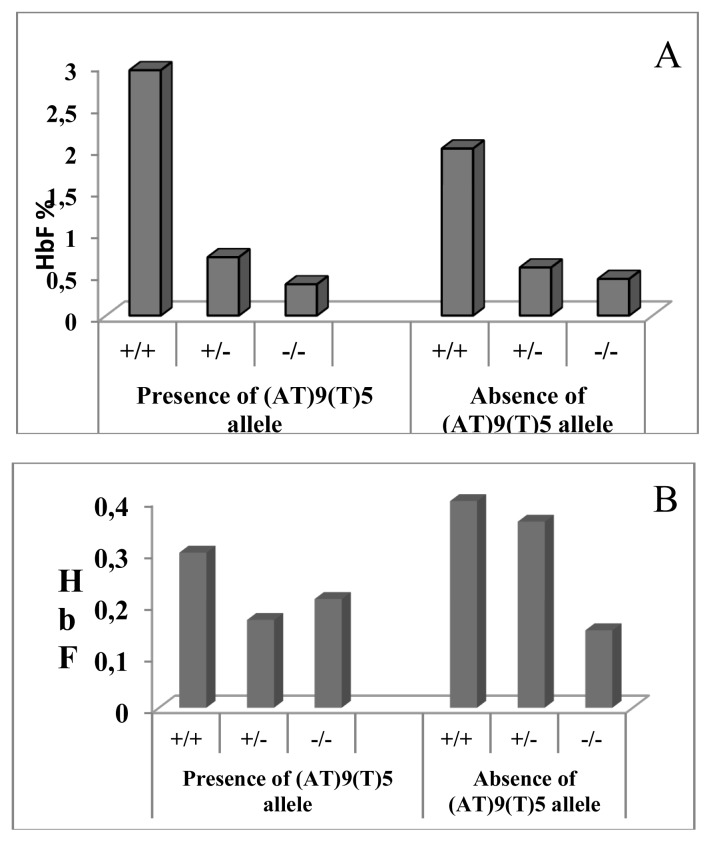
Association between combination of −158Gγ polymorphism, (AT)_9_(T)_5_ motif and HbF levels among (A) heterozygotes and (B) Normal control group.

In the individuals heterozygous for β thalassemia, we have found a strong association between moderately elevated HbF levels and presence of the T allele at −158 Gγ (Figure 1). 80% of the heterozygotes having HbF > 1.2% had shown the presence of *XmnI* polymorphism. Cases showing homozygosity for the T allele had HbF expression more than 2.2% (mean± SD 2.7± 0.45 %). Similar findings were reported by Guida et al (2006). In their study of 188 β thalassemia carriers and 229 wild type individuals of Italian descent, presence of the T allele contributed to increased levels of HbF in both the groups.^12^ In our study the normal control group did not show this correlation. Akbari et al (2008) reported that 60% of their thalassemia intermedia patients with β^0^/β^0^ genotype showed the homozygosity for the *XmnI* polymorphism (+/+) while the *XmnI* (−/−) genotype was present in homozygotes carrying β^+^/β^+^ genotype. Hence they concluded that the *XmnI* polymorphism seems to be one of the ameliorating factors of disease severity in Iranian population.^13^ Among our study, 50% of thalassemia intermedia cases showing β^0^/β^0^ genotype showed homozygosity for *XmnI* polymorphism (+/+). Nemati et al (2010) have studied the frequency of *XmnI* polymorphic site in beta-thalassemia major patients from Western Iran and has revealed that the presence of this polymorphic site caused a positive influence on Hb F production and the (G) gamma % which could improve the clinical symptoms of beta-thalassemia patients.^14^ Similar results are observed in our study.

There are several polymorphic repeat sequences within the β globin gene cluster. The DNA silencer region 5′ to the β globin gene acts as a negative regulatory element and it is possible that the β globin gene silencer region with the core structure (AT)_x_(T)_y_ may modulate expression of the β globin gene.^15^ Though we observed 3 different length polymorphisms: (AT)_7_(T)_7_, (AT)_9_(T)_5_ and (AT)_8_(T)_9_ among thalassemia patients, the (AT)_7_(T)_7_ motif seemed to be the prevalent configuration in normal individuals (80%). Both Bandopadhyay et al (2005) and Arya et al (2009) reported very high prevalence (65% and 100% respectively) of this motif in their normal population.^16^,^17^ The (AT)_9_(T)_5_ motif has been studied with varying implications. It has been associated with silent β thalassemia, a mild phenotype, and higher levels of HbF in some homozygous thalassemia patients. Some studies also demonstrated no direct association between any of the (AT)_x_(T)_y_ arrangements and an increased gamma gene expression (HbF) levels.^2^,^18^ Jouini et al (2012) evaluated the effect of 3 polymorphic markers [XmnI polymorphism, polymorphic repeats in the intron 2 of the G γ globin gene and the (AT)_x_(T)_y_ motifs] within β globin gene cluster on HBF expression in normal individuals of Tunisian population. They reported that 97% of the individuals having high levels of HbF carried one or more of these markers and suggested that the increase of HbF levels in healthy individuals is related to these polymorphisms.^19^

In our study among the β thalassemia heterozyotes only 5% of chromosomes carrying the (AT)_9_(T)_5_ configuration showed increase in HbF levels. The association between the combination of the −158G γ and (AT)_x_(T)_y_ polymorphism and increased HbF have been previously reported in β thalassemia trait and wild type subjects.^20^,^21^ In our study we found a trend of raised HbF in β thalassemia heterozygotes showing the presence of both the −158Gγ polymorphism and the (AT)_9_(T)_5_ configuration as against subjects showing absence of these two polymorphisms (Figure 1). No correlation was found between the motif configuration and HbF levels in the wild type subjects.

The data summarized in Table 1 indicates that a strong association exists between a specific β thalassemia mutation and the type of (AT)_x_(T)_y_ motif. Interestingly, all β thalassemia chromosomes carrying the Codon 15 (G→A) mutation showed the presence of (AT)_9_(T)_5_ allele. A study in Eastern Indian population had shown the linkage of known β thalassemia mutations with the (AT)_8_(T)_5_ configuration.^22^ Goncalves et al (1998) reported that presence of both the (AT)_9_(T)_5_ sequence configuration at position −530 of the β globin gene and a (C→T) variation at −158 of the G γ globin gene is associated with elevated expression of HbF. However, at least one defective β globin gene is required to reveal this association.^23^ Results of our study are in agreement with these findings.

Among our homozygous patients, 66% of the chromosomes from the thalassemia intermedia group showed the presence of the *XmnI* polymorphism. Similarly the (AT)_9_(T)_5_ motif was predominantly found in transfusion independent individuals (27%) as against transfusion dependent one (14%). Arya et al (2009) in their study on 20 North Indian β thalassemia homozygote patients with the IVS1 nt5 (G→C) mutation concluded that the *XmnI* polymorphism and the (AT)_x_(T)_y_ repeat motifs other than (AT)_7_(T)_7_ influence the phenotype by increasing the HbF expression and reducing the disease severity.^17^ Hence from these two cis acting regulators it becomes clear that an association exists between certain sequence configurations and the “High F” phenotype which might be contributing in ameliorating the disease severity.

An interesting study by Chan et al (2007) showed association between the (AC)_3_(AT)_7_ (T)_5_ configuration and raised HbF levels among the Chinese population.^24^ They suggested that (AC)_n_(AT)_x_(T)_y_ configuration may have an association with the HPFH phenotype which acts by suppression of the β globin gene expression. Berg et al (1991) have previously identified HMG-Y and HMG-1 protein which bind and bend the DNA at or near the BP1 binding site within the β globin gene promoter and facilitate the binding of BP1 and other repressor proteins in this region.^25^ Therefore BP1 and HMG protein may be important mediator proteins of β globin expression affecting the presentation of Sickle cell disease, β thalassemia and the HPFH phenotype.

## Figures and Tables

**Table 1:** Allelic distribution of (AT) x (T) y motifs with different β thalassemia mutations.

(AT)x(T)y motifs	β thalassemia Mutations										No. of Chromosomes
	IVS1-5 (G→C)	CD30 (G→C)	IVS1-1 (G→T)	CAP +1 (A→C)	CD41/42 (-CTTT)	CD15 (G→A)	−88 (C→T)	CD8/9 (+G)	CD86 (C→C)	619 bp del	
7/7	6(31%)	2(67%)	6(67%)	2(67%)	1(33%)	-	-	1	-	-	18
8/9	12(63%)	-	2(22%)	1(33%)	-	-	-	-	1	-	16
9/5	1(5.2%)	1(33%)	1(11%)	-	2(67%)	4(100%)	1	-	-	1	11
No. of Chromosomes	19	3	9	3	3	4	1	1	1	1	45

**Table 2:** (AT) x (T) y motif, Xmn1 polymorphism and HbF levels in 35 Thalassemia Major Patients.

Mutations(No.)	(AT)x(T)y polymorphism (No)	Xmn1 Polymorphism			HbF(%) at presentation
		+/+	+/−	−/−	

**IVS1nt5(G→C)+IVS1nt5(G→C) - (19)**	7/7/7/7 (3)	-	-	3	50–90
	8/9/8/9 (11)	2	4	5	55.8–99.3
	8/9/7/7 (2)	-	-	2	88.3–94.6
	8/9/9/5 (3)	-	1	2	60–88.7

**IVS1nt5(G→C) +CD 15 (G→A) - (2)**	8/9/7/7	-	1	1	78

**IVS1nt5(G→C)+CD 30 (G→C) - (2)**	7/7/7/7	-	-	2	55.4–75.5

**IVS1nt5(G→C)+ IVS1nt1(G→A) - (1)**	8/9/7/7	-	1	-	90.2

**IVS1nt1 (G→A)+IVS1nt1(G→A) - (1)**	7/7/7/7	1	-	-	70.6

**619 bp del +IVS1nt1(G→A) - (3)**	8/9/9/5	-	-	3	55.7–88.9

**619 bp del+ 619 bp del - (2)**	8/9/7/7	-	-	1	80.9
	7/7/7/7	1	-	-	85.7

**CD41/42(-CTTT)+ CD41/42(-CTTT) - (1)**	9/5/9/5	-	-	1	95.3

**IVS1nt5(G→C)+CD41/42(-CTTT) - (1)**	8/9/9/5	-	-	1	97.5

**IVS1nt1(G→A)+ CD41/42(-CTTT) - (1)**	7/7/9/5	-	-	1	97.4

**FS8/9 (+G) + Capsite +1(A→C) - (1)**	7/7/7/7	-	-	1	37.8

**IVS1nt5(G→C) +CD 5(-CT) - (1)**	8/9/9/5	-	-	1	50.6

**Table 3:** (AT) x (T) y motif, Xmn1 polymorphism and HbF levels in 15 Thalassemia Intermedia patients.

β thalassemia Mutation	Age(yrs)	Age at presentation(yrs)	Transfusion frequency	XmnI polymorphism	(AT)x(T)y	HbF (%)
**IVS1nt5(G→C)+IVS1nt5(G→C)**	3	3	UT	+/−	7/7/7/7	92.8
**IVS1nt5(G→C)+IVS1nt5(G→C)**	14	10	UT	+/+	7/7/7/7	76.5
**IVS1nt5(G→C)+IVS1nt5(G→C)**	6	5	UT	+/+	8/9/8/9	94.6
**IVS1nt5(G→C)+IVS1nt5(G→C)**	18	12	Intermittently	+/+	8/9/8/9	83.0
**IVS1nt5(G→C)+Codon30(G→C)**	7	2	UT	+/−	7/7/9/5	85.4
**FS41/42(-CTTT)+Capsite+1(A→C)**	20	14	UT	−/−	7/7/9/5	53.8
**CD15(G→A)+CD15(G→A)**	5	3	UT	+/+	9/5/9/5	98.1
**CD30(G→C)+CD30(G→C)**	13	12	UT	+/+	7/7/7/7	97.4
**IVS1nt1(G→A)+IVS1nt1(G→A)**	4	3	UT	+/+	7/7/9/5	97.1
**IVS1nt1(G→A)+619 bp del**	17	4	Monthly	+/−	7/7/7/7	11.6
**IVS1nt1(G→A)+619 bp del**	7	4	UT	+/−	7/7/9/5	87.3
**IVS1nt1(G→A)+CD8/9(+G)**	13	5	7 times	+/−	7/7/7/7	98.3
**CD15(G→A)+Capsite+1(A→C)**	9	9	UT	+/−	9/5/7/7	63.4
**−88(C→T)+ IVS1nt1(G→A)**	2	2	UT	+/−	7/7/9/5	97.6
**−86(C→G) +IVS1nt5(G→C)**	4	4	UT	+/−	7/7/7/7	93.5

UT - Untransfused
